# Comprehensive assessments and related interventions to enhance the long-term outcomes of child, adolescent and young adult cancer survivors – presentation of the CARE for CAYA-Program study protocol and associated literature review

**DOI:** 10.1186/s12885-019-6492-5

**Published:** 2020-01-06

**Authors:** J. Salchow, J. Mann, B. Koch, J. von Grundherr, W. Jensen, S. Elmers, L. A. Straub, E. Vettorazzi, G. Escherich, S. Rutkowski, S. Dwinger, C. Bergelt, M. Sokalska-Duhme, S. Bielack, G. Calaminus, K. Baust, C. F. Classen, C. Rössig, J. Faber, H. Faller, I. Hilgendorf, J. Gebauer, T. Langer, M. Metzler, S. Schuster, C. Niemeyer, A. Puzik, D. Reinhardt, U. Dirksen, A. Sander, M. Köhler, J. K. Habermann, C. Bokemeyer, A. Stein

**Affiliations:** 1 University Medical Center Hamburg-Eppendorf, Hamburg, Germany; 2 Klinikum Stuttgart, Olgahospital, Stuttgart, Germany; 3 University Hospital Bonn, Bonn, Germany; 4 University Hospital Rostock, Rostock, Germany; 5 University Children’s Hospital Münster, Münster, Germany; 6 Mainz University Medical Center, Mainz, Germany; 7 University Hospital Würzburg, Würzburg, Germany; 8 University Hospital Jena, Jena, Germany; 9 University Hospital of Schleswig-Holstein, Campus Lübeck, Lübeck, Germany; 10 University Hospital Erlangen, Erlangen, Germany; 11 Medical Centre, Faculty of Medicine, University of Freiburg, Freiburg, Germany; 12 University Hospital Essen, Essen, Germany; 13 German Cancer Consortium, Essen, Germany; 14 Hannover Medical School, Hannover, Germany; 15 Medical Faculty University Hospital Magdeburg, Magdeburg, Germany; 16 University of Lübeck, Lübeck, Germany

**Keywords:** CAYA, AYA, Children adolescents and young adults after cancer, Long-term toxicity, Long-term side effects, Lifestyle intervention, Prevention programme, Follow-up care

## Abstract

**Background:**

Improved, multimodal treatment strategies have been shown to increase cure rates in cancer patients. Those who survive cancer as a child, adolescent or young adult (CAYA), are at a higher risk for therapy-, or disease-related, late or long-term effects. The CARE for CAYA-Program has been developed to comprehensively assess any potential future problems, to offer need-based preventative interventions and thus to improve long-term outcomes in this particularly vulnerable population.

**Methods:**

The trial is designed as an adaptive trial with an annual comprehensive assessment followed by needs stratified, modular interventions, currently including physical activity, nutrition and psycho-oncology, all aimed at improving the lifestyle and/or the psychosocial situation of the patients. Patients, aged 15–39 years old, with a prior cancer diagnosis, who have completed tumour therapy and are in follow-up care, and who are tumour free, will be included. At baseline (and subsequently on an annual basis) the current medical and psychosocial situation and lifestyle of the participants will be assessed using a survey compiled of various validated questionnaires (e.g. EORTC QLQ C30, NCCN distress thermometer, PHQ-4, BSA, nutrition protocol) and objective parameters (e.g. BMI, WHR, co-morbidities like hyperlipidaemia, hypertension, diabetes), followed by basic care (psychological and lifestyle consultation). Depending on their needs, CAYAs will be allocated to preventative interventions in the above-mentioned modules over a 12-month period. After 1 year, the assessment will be repeated, and further interventions may be applied as needed. During the initial trial phase, the efficacy of this approach will be compared to standard care (waiting list with intervention in the following year) in a randomized study. During this phase, 530 CAYAs will be included and 320 eligible CAYAs who are willing to participate in the interventions will be randomly allocated to an intervention. Overall, 1500 CAYAs will be included and assessed. The programme is financed by the innovation fund of the German Federal Joint Committee and will be conducted at 14 German sites. Recruitment began in January 2018.

**Discussion:**

CAYAs are at high risk for long-term sequelae. Providing structured interventions to improve lifestyle and psychological situation may counteract against these risk factors. The programme serves to establish uniform regular comprehensive assessments and need-based interventions to improve long-term outcome in CAYA survivors.

**Trial registration:**

Registered at the German Clinical Trial Register (ID: DRKS00012504, registration date: 19th January 2018).

## Background

### Epidemiology

Roughly 500,000 people receive a new cancer diagnosis every year in Germany, of which 2200 (0.4%) are under the age of 18 and 16,000 (3.0%) are between the ages of 19 and 39.^1^ This relatively small group of cancer patients under the age of 39 are called “CAYAs” (children, adolescents and young adults).^2^ While there are many differences among this group, including: the heterogeneity of cancer diagnosis, treatment protocol, current life situation; they also have a lot in common, for example the relatively high cure rates (> 80%), and aggressive multimodal treatment that increase the risk of long term sequelae [1–3].

The most commonly diagnosed cancers in adolescent and young adult women (15 to 39 year old) are breast cancer (28%), melanoma (16%), thyroid cancer (11%) and cervical cancer (10%) [3]. While in 15 to 39 year old men, germ cell tumours (34%), melanoma (11%), Hodgkin’s lymphoma (8%) and non-Hodgkin’s lymphoma (6%) are the most prevalent cancers [4]. In children (under the age of 15) leukaemia (33%), brain tumours (24%) and lymphoma (11%) are predominantly diagnosed [3, 4].

### Long-term sequelae in CAYA cancer survivors

Cancer treatment may cause immediate side effects occurring during or directly after treatment (e.g. haematological or gastrointestinal toxicities), which are generally detected immediately and treated with the respective supportive measures. However, the treatment may also cause late effects, which may not become apparent until years or even decades later (e.g. cardiac toxicities or secondary cancers). The Childhood Cancer Survivor Study (CCSS) utilizing a long-term follow-up in 10,397 CAYAs found that two out of every three CAYAs have at least one treatment-related long-term toxicity with one out of three CAYAs developing a severe or life-threatening late effect [5]. Disease or treatment related long-term toxicities may affect any organ, e.g. heart, lungs, gastrointestinal tract, kidneys and bladder, skin, eyes, brain, bones or the endocrine and reproductive systems, and are not necessarily confined to the organ of the original cancer diagnosis [5–7]. Furthermore, psychosocial issues, for example the fear of recurrence, fear and anxiety concerning their future, depression, post-traumatic stress disorder (PTSD), long-term educational and work problems or social and behavioural difficulties are common problems [5, 8, 9].

#### Physical long-term sequelae

The most commonly reported long-term toxicities in cancer survivors are cardiovascular diseases like cardiomyopathy, chronic heart failure or valvular disorder, which occur with a five to 15-fold frequency, and at an earlier age, when compared to the general population [5, 10]. The individual risk for the development of cardiovascular disease is determined by treatment related factors (e.g. type, mode of administration and cumulative dose of chemotherapy and/or chest radiotherapy), and non-treatment related factors like lifestyle (e.g. smoking) or relevant co-morbidities (e.g. dyslipoproteinaemia or hypertension). Chest-directed radiotherapy is associated with an increased risk of myocardial infarction, congestive heart failure, valvular heart disease, and arrhythmias [11]. Anthracycline chemotherapy increases the risk of heart failure [11, 12]. CAYAs exposed to prior anthracycline-based treatment and chest radiation have the highest treatment-related risk for cardiovascular diseases. Thus, aftercare focusing not only on tumour relapse or second cancer, but also improving modifiable lifestyle risk factors is of particular importance.

CAYAs are more often obese when compared to siblings, especially after hypothalamic injury due to resection, radiotherapy or high doses of corticosteroids (e.g. after brain cancer or acute lymphoblastic leukaemia (ALL) treatment) [13, 14]. High incidence rates of diabetes mellitus and insulin resistance (roughly 50%) are reported after allogeneic haematopoietic stem cell transplantation (HSCT) or abdominal radiotherapy for solid tumours [15, 16].

Up to one in five CAYAs have problems with decreased bone mineral density due to the direct impact of the cancer itself (e.g. leukaemia), corticosteroid treatment, osteotoxic chemo- and/or radiotherapy, treatment-induced endocrine disorders (e.g. growth hormone deficiency or hypogonadism), malnutrition, physical impairment or reduced muscle strength [17–19]. These aforementioned long-term effects may influence the lifestyle of CAYAs and therefore increase the risks of long-term side effects like cardiovascular diseases.

#### Psychological and social sequelae

Due to the disturbance of the psychosocial development period during childhood, adolescence and young adulthood, CAYAs are particularly vulnerable to psychosocial problems [20]. Although a cancer diagnosis clearly impacts the psychosocial situation at every age, the CAYA age is a critical period in life. Establishing identity, developing a sexual identity and a positive body image, as well as separating from parents, being around peers and (starting to) make decisions regarding career and employment, education and family are the typical concerns of young people transitioning from childhood to adulthood [21–23]. Therefore, cancer and cancer-related issues (e.g. confrontation with mortality, changes in body image, dependence on parents, disruptions in social life and education / employment, loss of reproductive capacity) can be more stressful for cancer survivors than for healthy young adults [21, 22, 24]. Therefore, compared to the general population, the risk for behavioural and educational problems are twice as high; and quality of life, mental well-being and life satisfaction are much lower in CAYAs with cancer [25].

CAYAs often have difficulties with reintegration into school, work, education and everyday life that may lead to missing graduation and financial problems. Furthermore, not all cancer survivors are able to return back to work or school at all [26]. About 72% of patients who were working, or in school, full-time before diagnosis returned to full-time work or school 15 to 35 months post diagnosis, but only 34% of previously part-time workers/students and 7% of homemakers returned back [26]. In addition, young adult survivors of childhood allogeneic HSCT have high unemployment rates at all attained ages (18–22 (56%), 23–37 (53%) and 28–32 (68%) years) [27].

When compared to the general population, CAYAs have more educational or other school problems (46% vs. 23%), including having to repeat a grade (21% vs. 9%) and developing a learning-disability (19% vs. 7%) or having to attend special-education programmes (20% vs. 8%) [27]. CAYAs with central nervous system (CNS) tumours or leukaemia receiving CNS radiation are at a particularly high risk for problems at school [28]. In addition, cancer history may influence social relationships and interactions. CAYAs tend to have less close friends (19% vs. 8%) and were less likely to use friends as confidants (58% vs. 67%) when compared to peers [28]. Young adult cancer survivors are more likely to divorce or separate than same-age controls [29]. Nearly 50% of CAYAs have reported financial distress, annual productivity loss, or debt accumulation due to treatment costs, or did not adhere to recommended prescription medication because of the uninsured costs [30].

Furthermore, survivors of childhood cancer were at high risk for hospitalization, and spent an average of five times more days in hospital, when compared to controls [31]. Major reasons for hospitalization among cancer survivors include diseases of the nervous system (19.1% of all excess hospitalizations), endocrine system (11.1%), digestive organs (10.5%) and respiratory system (10.0%) [31].

#### Lifestyle and risky health behaviour of cancer survivors

Although CAYAs have faced a severe life threatening disease in their early years, up to 35.8% of survivors will develop a risky health behaviour (sexual behaviour, tobacco, alcohol, or illicit drugs) [32]. However, data comparing the risky behaviour to siblings or the general population remain inconsistent. Some studies report that cancer survivors smoke, consume alcohol and use illicit drugs at rates lower than siblings [33], but other studies found no difference or increased risky health behaviour among AYA survivors of childhood cancer [34, 35]. A recent meta-analysis of the available literature showed that 22% of survivors smoked, 20% were binge drinkers, and 15% used drugs [36].

In addition to risky behaviour, survivors tend to have an unhealthy lifestyle, with only 10% following a healthy lifestyle [37]. A large number of cancer survivors are overweight (58%), eat less than the recommended five servings of fruits and vegetables per day (82%) or fail to do any sport activities (55%) [37]. In the INAYA1 (“Improved Nutrition in AYAs”) trial, 74 and 22% of CAYAs had a moderate and bad nutritional behaviour, respectively [38]. Similar results were shown in the INAYA2 trial with 66 and 14% having a moderate or bad nutrition behaviour (presentation DGHO 2018) [38]. Additionally, 15% of CAYAs consume an excessive amount of salt (≥ 10 g per day). Both studies showed that only a few childhood cancer survivors met the nutrition recommendations of the German Nutrition Society (DGE) (www.dge.de/10regeln). Similar results were found in American childhood cancer survivors whose mean HEI-2010 was about 50% of the maximum score [39]. Interestingly, long-term survivors (time from diagnosis ≥10 years) had a significantly lower HEI-2010 than recent survivors (time from diagnosis < 5 years) (*P* = 0.047). CAYAs struggle to adhere to the consumption of green vegetables and beans, total vegetables and whole fruits. No survivor met the guidelines for dietary fibre and potassium intake and only a few met the guidelines for vitamin D, sodium, calcium, and saturated fat intake. The average for saturated fat and for sodium was 115 and 143% respectively [39].

Another relevant factor of a healthy lifestyle is regular physical activity. Previous studies have shown, that CAYAs were insufficiently active compared with controls [40–42] and had a low motor performance at the end of the acute treatment phase [43], with serious reductions in motor performance within two years after bone tumour treatment. The positive impact of physical activity on the risk for long-term sequelae, has been shown in a variety of retrospective studies, with very few focusing on CAYAs. In HSCT survivors, correlations between increased physical activity levels (endurance) and lower waist circumference, lower percent fat mass and greater insulin sensitivity were noted [44]. A prevalent and distressing symptom in children and adolescents with cancer, and in those who have undergone HSCT, is fatigue. A multidisciplinary group of experts in paediatric oncology and fatigue, developed a clinical practice guideline for management of fatigue with the focus on physical activity, relaxation and mindfulness [45].

A report from the CCSS noted that Hodgkin’s lymphoma survivors (median, age 31.2 years) regularly undergoing vigorous exercise (≥ 9 metabolic equivalent [MET] hours/week [h/wk]) had a significantly lower risk of treatment-related cardiovascular events than survivors not meeting the guidelines for vigorous intensity exercise. For survivors who reported ≥9 MET-h/wk., the cumulative incidence of any cardiovascular event was 5.2% at ten years from baseline. In comparison, the cumulative incidence for survivors who reported 0 MET-h/wk. had more than doubled to 12.2% [46]. By analysing 15,450 adult cancer survivors (median, age 25.9 years) from the CCSS cohort, at 15 years from baseline, the increase in vigorous exercise over an eight-year period was associated with a significant reduction of 40% in the risk of all-cause mortality, when compared to the survivors who only maintained low levels of exercise (3 to 6 MET-h/wk) [47].

#### Lifestyle interventions

Improving lifestyle behaviour is key to reducing the risk for cardiovascular long-term toxicities in particular. Given the fact that a sedentary lifestyle, lack of physical activity and poor nutrition increase the risk factors for cardiovascular diseases [48], there is an unused opportunity to improve the young cancer survivors’ risk profile. Thus, several interventional trials focused on CAYAs have since been undertaken. The INAYA1 trial aimed to evaluate the feasibility and the impact of an intensified nutrition counselling programme targeted to the at risk subsection of CAYAs [38]. Nutritional behaviour was improved in week 12 by intensified nutrition counselling and a good, moderate and bad nutritional intake was seen in 48, 52 and 0% of CAYAs, compared to 4, 74 and 22% at baseline, respectively. No clinically relevant improvement was seen in quality of life, Waist-Hip Ratio (WHR), Body Mass Index (BMI) and blood pressure. The consecutive INAYA2 trial was able to show a decrease of sodium intake. Despite the INAYA trials, there is still a lack of nutritional interventions for young cancer survivors. The Survivor Health and Resilience Education (SHARE) Program focused on bone health behaviours among adolescent survivors of childhood cancer (median, age 14.2 years). This intervention had a significant short-term impact at one-month follow-up. Compared with the control group, participants of the intervention group had higher milk consumption, calcium supplementation and dietary calcium intake [49]. Another double-blind randomized controlled trial (median age 17 years) focusing on bone health of long-term survivors of childhood ALL used calcium and cholecalciferol supplementation (or a placebo). This trial came to the conclusion that cholecalciferol and calcium supplementation provided no additional benefit to nutritional counselling for improving lumbar spine bone mineral density among adolescent and young adult survivors of ALL [50].

Regarding the physical activity of CAYAs, only a few randomized controlled trials with very small sample sizes exist so far. These studies can be classified in three main categories: home-based, web-based or supervised physical activity interventions. A home-based intervention with asymptomatic childhood acute lymphoblastic leukaemia survivors included a three-month exercise programme and reported an improved cardiac function, in terms of a significant improvement of the attenuated left ventricle diastolic function [51]. Another home-based intervention where participants met physical activity guidelines and wore a motivational activity tracker over a six-month period led to an increased, but not statistically significant, moderate to vigorous physical activity and maximum oxygen uptake (VO_2 max_) [52]. A similar intervention focusing on a ten-week home-based exercise programme with feedback from a pedometer, and supported by a counsellor, led to a significant decrease in fatigue and significant increase in daily physical activity (steps per day) [53]. Online interventions focused on promoting health behaviour via email over a six-week period [54] or using a physical activity website for 12 weeks [55]. Though, these studies determined high feasibility and acceptability, physical activity levels did not change or increase significantly. A Facebook-based physical activity intervention over a three-month period increased moderate to vigorous physical activity and led to significant weight loss [56]. Supervised interventions containing a physical activity-educational and/or exercise intervention in a group setting improved physical activity, quality of life and also cardiovascular, physical and metabolic outcomes of cardiovascular diseases [57, 58].

In our clinic we conducted the MAYA trial (Motivate AYA, presentation DGHO 2018, publication in process), where we randomly evaluated the effect of a structured intervention on physical activity and quality of life in CAYAs with cardiovascular risk factors. CAYAs of the intervention group increased the amount of vigorous intensity activity from baseline to week 12 and reduced the amount of time spent sitting.

#### Psycho-oncological interventions

Several behavioural intervention techniques are used to address mental distress in cancer survivors, including [59] the transtheoretical Model (TTM), cognitive behavioural therapy (CBT) and motivational interviewing (MI). Current literature is still inconclusive as to which of these show the best effect [60]. MI seems to be a promising approach as it targets patients that feel ambivalent about a certain behaviour, knowing on the one hand about the disadvantages, and on the other hand seeing the benefits of said behaviour. Therefore it is compatible with a variety of problems that CAYAs feel ambivalent about, such as classic health behaviours like smoking cessation, alcohol consumption, physical activity and nutrition. Although initially developed to address addiction, nowadays MI is widely used across the medical field to address a broader range of behaviours [61]. MI uses reflective listening and a client-centred approach to help the patient explore their own motivation to change and their way of planning and realizing said changes. Further techniques used in MI are the expression of empathy, the development of discrepancies between the actual behaviour and the patients’ goals, the avoidance of confrontation within the therapeutic relationship and the enhancement of optimism and self-efficacy [62]. Therefore, CAYA specific topics, like changing their way of coping with cancer, of dealing with fear of reoccurrence or of coping with fatigue symptoms may also be addressed using MI techniques, despite the fact that scientific evidence in this regard is sparse.

The existing evidence regarding MI in cancer survivors seems promising: Spencer et al. [61] included 15 studies using MI in cancer survivors in their systematic review. They concluded that MI techniques seem to be effective - besides impacting health behaviours like nutrition and activity, MI may decrease patient stress related to cancer [63, 64] and may enhance overall quality of life [65–67]. Regarding fatigue and pain the evidence remains inconclusive.

#### Survivorship programmes for CAYAs

Follow-up care of CAYAs is challenging in itself, as it encompasses more than detection of cancer relapse programmes, which are necessary but so far rarely available 67% of CAYAs have no access to specialised CAYA aftercare [68]. In the United States of America, patients post cancer are treated in survivorship clinics, but sadly there is no such centralized institution in Germany or Europe. Examples of prevention or support programmes for CAYAs in Germany include: OncoKids (www.neu.onko-kids.de), the Phönikks foundation (www.phoenikks.de), the Pancare network (www.pancare.eu/en), AYA parents (www.khae.ovgu.de/SAYA.print), JET trial (www.uniklinikum-jena.de), AYALE trial (www.uniklinikum-leipzig.de) and “Deutsche Stiftung für junge Erwachsene mit Krebs” (www.junge-erwachsene-mit-krebs.de). Programmes for young cancer survivors with the focus on lifestyle, health behaviour, particularly with regard to healthy diet and regular physical activity, are lacking.

Also lacking are necessary conclusions, data about treating or preventing long-term effects, which are also heterogeneous and generally incomparable. There is a lack of randomized controlled trials dealing with the topic of our paper. A standardized follow-up care programme of CAYAs in Germany does not exist, especially with a focus on the long-term consequences of cancer survivorship. Based on the results of the aforementioned interventional trials, there is a dire need to establish a regular and comprehensive assessment, and related interventions, covering preventative lifestyle and psychological issues. This paper presents the first structured and randomized follow-up programme focusing on lifestyle and psychological consequences and appropriate interventions in CAYAs.

## Methods/design

**Fig. 1:**
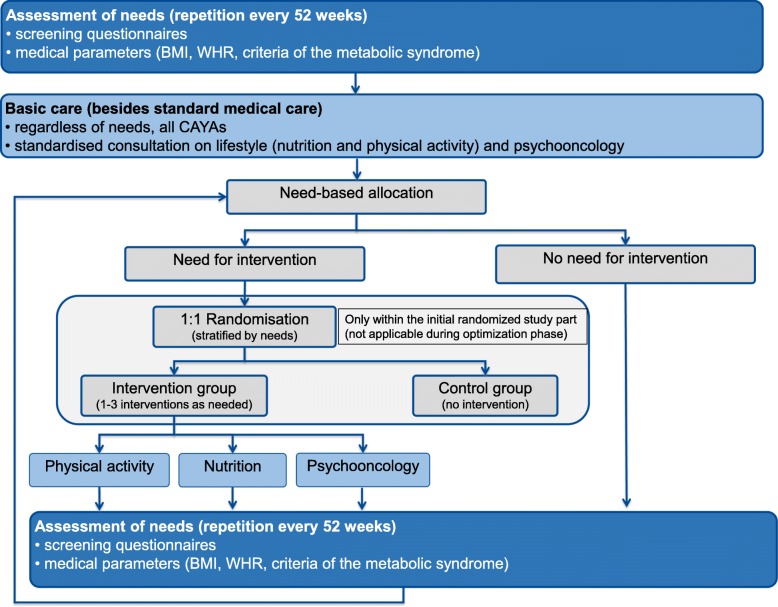
Flow chart of the CARE for CAYA-Program

Based on the physical, psychological and social long-term sequelae of CAYAs, the current literature and our experience in our survivorship clinic, we designed the CARE for CAYA-Program (CFC-P). This programme was designed to be an adjunct to the medical follow-up care, with the aim of assessing the needs of CAYA survivors and applying need-based interventions to prevent potential long-term sequelae. Thus the CFC-P includes annual comprehensive assessments to determine the individual need for a single, or several, preventative intervention(s) (high need) or no need for a preventative intervention (low need), followed by need-stratified modular interventions, currently including physical activity, nutrition, and psycho-oncology (Fig. 1). The CFC-P was developed and is currently conducted in a consortium of 15 sites in Germany with established follow-up care clinics for CAYAs.

The program is running and will be implemented in addition to a survivorship clinic run by medical doctors, who are focusing on medical issues regarding either cancer recurrence or medical long-term effects. Within these established structures no nutritional, physical activity or psycho-oncological support is integrated or reimbursed on a general basis yet, particularly not with preventive intention (not treating a pre-existing disorder).

The CFC-P will be conducted within the framework of the innovation fund of the German Federal Joint Committee and thus aims to establish the efficacy of the programme with a randomized trial, followed by the implementation into the general care including the potential reimbursement of the interventions. Therefore, the programme will continue after completion of the randomization phase, and further evaluations regarding the assessment and the interventions will be conducted. Within the innovation fund, projects are limited to an overall duration of three years, thus the assessment of short-term effects was chosen to determine the efficacy of the programme.

### Need-stratified assessment

The CFC-P includes a need-stratified assessment for the three modules: physical activity, nutrition, and psycho-oncology. The screening for need within the physical activity module is based on a questionnaire that was specially developed for the programme, because an adequate pre-existing questionnaire was not found that could be used for screening in this population. It includes questions regarding an average week within the past month: *1. On how many days in an average week have you been physically active at a moderate intensity? How long have you been physically active on these days?* And *2. On how many days in an average week have you been physically active at a vigorous intensity? How long have you been physically active on these days?* CAYAs who are less active than 150 min of moderate, or 75 min of vigorous intensity (or a combination of both intensities), or indicated activity on less than three days a week, are classified as having a need for an intervention.

Within the nutrition module the CAYAs filled in a three-day dietary record (“Freiburger Ernährungsprotokoll”) [69] providing data to calculate the “Healthy Eating Index- European Prospective Investigation into Cancer and Nutrition” (HEI-EPIC) [70]. HEI-EPIC is an established instrument to evaluate dietary behaviour [71]. In the study the validated German version of HEI, the HEI-EPIC was used [70]. This instrument has been used within the INAYA trial and was considered as appropriate for this population [38]. The HEI-EPIC distinguishes the following eight food groups: drinks, vegetables, fruits, cereals/potatoes, milk/dairy products, meat/sausages/fish/eggs, fats/oil and sweets/snacks. Based on a calculation described by Rüsten et al. 0–10 points for each group of food with up to 20 points for fruits, vegetables and drinks were calculated [70]. The sum score ranges from 0 to 110 points. A sum score ≤ 40 points indicate a bad, > 40–64 points a moderate and ≥ 65 points a good dietary behaviour [70, 72]. CAYAs with a HEI EPIC score of ≤40 are in need of a nutrition intervention.

For the modules physical activity and nutrition there are further criterions for a need defined, for example meeting the criteria for metabolic syndrome (Table 2).

For the psycho-oncology module, the needs assessment consists of the German version of the NCCN Distress Thermometer [73]. It consists of a general scale scored from 0 to 10, as well as an additional problem list. As a score of five is internationally recognized as an indicator that a patient is distressed and needs support, this is also used as a cut-off for the psycho-oncology module. For a score of five in the Distress-Thermometer, Mehnert et al. found a sensitivity of up to 84% and a lower specifity of up to 47% when screening for moderate levels of anxiety or/and depression with the Hospital Anxiety and Depression Scale (HADS-D). The second screening instrument for this module is the German version of the Patient Health Questionnaire (PHQ-4) [74]. The Chronbach’s α = 0.82 showed good internal consistency, and construct validity of the PHQ-4 was supported by intercorrelations with other self-reported scales [68].

### Modular interventions

The three modules will be conducted by therapeutic personnel (e.g. sport scientists, physiotherapists, dieticians or nutrition scientists, psycho-oncologists) and follow a stringent interview guide. For every module a comprehensive manual was formulated, which was applied in each CFC site. Also, the personnel in every site was trained at the beginning of the programme and participated in regular telephone conferences.

The physical activity module includes five consultation hours within six months. The intention of the consultation is to motivate the CAYAs to increase their physical activity, especially that of vigorous intensity. Based on the TTM, individual objectives will be determined and possible barriers for not being active will be identified [75, 76]. In addition to the five consultations, the participants receive newsletters with general information about physical activity, and also individual newsletters.

The nutritional counselling includes five consultation hours within six months. The consultations are based on the standardized German nutrition care process including nutrition assessment, nutrition diagnosis, nutrition intervention and nutrition monitoring and evaluation [77]. The nutritionist is giving individual advice for a healthy diet to prevent a relapse and is helping the CAYAs to identify the barriers which prevent them from eating healthy and how to overcome these. In addition to the five consultations the CAYAs receive general and individual newsletters and are invited to a shopping training and a cooking class in order to support a healthy diet.

The psycho-oncology module includes five sessions of MI, on an approximately biweekly schedule. MI is a patient centred and guided approach of therapeutic communication with the goal of enhancing a person’s self-motivation in order to reach their goals by changing their behaviour. In the initial session the patient and therapist will select a focus for the next sessions. The sessions take 50 min and will be run by a certified psycho-oncologist, trained in MI. Regular telephone supervision will be provided by a senior psycho-oncologist and a certified M.I. trainer.

### Hypotheses

There are two primary hypotheses of the CFC-P, one focused on evaluating the interventions themselves and one focused on evaluating the assessment process. In this respect, it is expected that the adaptive interventions of the CFC-P will improve the lifestyle (nutrition and/or physical activity) and/or the psychological situation of the participants. Additionally, the evaluation and the adaptation of the annual assessment schedule will improve the coverage of unmet needs of CAYAs. Secondarily, the CFC-P should prove to be a feasible and cost-effective programme, as it utilises an adequate and effective needs-adapted participant allocation scheme. This, in combination with the effective interventions, will improve the cardiovascular risk profile and quality of life of CAYAs.

### Endpoints

Primary Endpoint of the CFC-P

- Rate of CAYAs with need for intervention after 12 months (rate in %, defined as CAYAs with need for intervention/ all in the trial included CAYAs) in comparison to the intervention and control groups in the randomized study part).

Co-primary Endpoint of the CFC-P

- Rate of CAYAs with unmet needs that are outside of the scope of the assessment (comparison of initial assessment and adapted assessment).

Secondary endpoints of the CFC-P

- Feasibility (recruitment, completion of assessments, adherence to and dropout rates of the overall programme and the respective interventions)
- Cost effectiveness (secondary health care costs, health care utilizations)
- Allocation and efficacy of modular interventions (difference in the individual need, cardiovascular risk factors and quality of life or fatigue at 12 months, in relation to the initial assessment and the participation in an interventional module

Additionally, the intervention modules will be assessed separately by applying specific endpoints for each module to assess the efficacy of the respective intervention after 12 months. To detect potential short-term effects, which may attenuate over time, an additional assessment will be performed after four months. These endpoints include changes in the respective questionnaires or in the objective parameters (e.g. BMI, phase angle in bioelectrical impedance analysis or spiroergometry).

### Inclusion criteria

Patients between the age of 15 and 39 years who received treatment for their cancer as a CAYA and are tumour free and in follow-up care will be included.

### Course of the programme (Fig. 1 flow chart)

At baseline (and subsequently on an annual basis) the current medical, psychosocial situation and lifestyle will be assessed from all included CAYAs. The assessment will be completed using validated questionnaires (e.g. EORTC QLQ C30, NCCN distress, PHQ-4, BSA, HEI-EPIC) and objective parameters (e.g. BMI, WHR, hyperlipidaemia, hypertension, diabetes).

All participants will receive a psychological and lifestyle consultation immediately after the assessment as basic care. Based on their individual needs, CAYAs with low needs will be reassessed after one year, whereas those with high needs will be allocated to a single, or several, preventative interventions (module) as needed (Table 1). The assessment will be repeated annually, and further preventative interventions may be applied.

**Table 1:** Module and interventions

Module	Schedule	Intervention / Assessment
Physical activity	week 0	• baseline assessment
	5 consultations á 60 min at week 0, 6, 12, 18, 24 +/− 4 weeks	• individual advices (consultation hour) about physical activity
	measurements at week 0, 16 and 52 +/− 4 weeks	• wearable activity monitoring over one week (ActiGraph) • bio impedance analysis (BIA)
	measurements at week 0, 16 and 52 +/− 4 weeks	• spiroergometry (additional measurement center-specific)
	in between week 0–52 every 6 weeks +/− 4 weeks	• newsletter with information’s about healthy nutrition and physical activity
Nutrition	week 0	• baseline assessment
	5 consultations á 60 min at week 0, 6, 12, 18, 24 +/− 4 weeks	• individual advices (consultation hour) about healthy diet
	measurements at week 0, 16 and 52 +/− 4 weeks	• bio impedance analysis (BIA)
	measurements at week 0, 16 and 52 +/− 4 weeks	• anamnesis of smell and taste; taste test (additional measurement center-specific)
	between week 8 and 20 +/− 4 weeks	• purchasing training (of 45–60 min) within a group of max 5 participants
	between week 8 and 20 +/− 4 weeks	• cooking class (of 2–3 h) within a group of max 8 participants
	in between week 0–52 every 6 weeks +/− 4 weeks	• newsletter with information’s about healthy nutrition and physical activity
Psychooncology	5 appointments à 50 min (individual date arrangement)	• ono-to-one session: motivational interviewing
	in between week 0–52 every 6 weeks +/− 4 weeks	• newsletter with information’s about coping, self-care and mental health

In the initial randomized phase, CAYAs with high needs will be randomized between preventative modular interventions (nutrition, physical activity and/or psycho-oncology) over a 12-months period, or basic care (waiting list, option to participate in the second year).

Every 12 months all CAYAs will get a tablet-based screening form including the following validated and objective questionnaires:

- NCCN Distress Thermometer (DT) [73]
- EORTC QLQ-C30 [78]
- 3-day dietary record (Freiburger Ernährungsprotokoll) [69]
- Modified Physical Activity, Exercise and Sport Questionnaire (BSA) supplemented with the Borg-Scale [79, 80]
- Patient Health Questionnaire (PHQ-4) [74]
- Questions about unmet needs
- Modified questionnaire about satisfaction (ZUF-8) [81]
- Measure of health status (EQ. 5D-5 L) [82]
- Questionnaire about schools, work [83]
- Questionnaire about loss of working hours [84]
- Short questionnaire about of use medical services [85, 86]
- SCNS-TF-9 [87]

Depending on the answers all patients will be classified in two groups. Group one will be those patients with a high need for intervention in at least one module and group two are patients without need for intervention.

Criteria for high need for intervention are set separately for each module and can be found listed in Table 2.

**Table 2:** Criteria for need of modular intervention

Module	High need for intervention (at least on criteria)
Physical activity	• < 150 min/week moderate physical activity and/or < 75 min intensive physical activity or • < 3 days physical activity per week or • criteria of metabolic syndrome
Nutrition	• ≤ 40 HEI-EPIC-Score or • BMI < 18.5 or > 25 or • WHR in women > 0.85, WHR in men > 1.0 or • gastrointestinal symptoms (e.g. diarrhea, nausea and vomiting, pain) or • criteria of metabolic syndrome
Psychooncology	• ≥ 6 points PHQ-4 • ≥ 5 points NCCN-DT

### Randomization and blinding

The annual comprehensive assessment will be performed after study inclusion by the responsible study personnel in each site for the three modules (physical activity, nutrition and psycho-oncology). The wearable activity monitoring over one week (ActiGraph) will be evaluated electronically and the bio impedance analysis (BIA) will be performed with standardized criteria to avoid any bias. When a high need in one of the modules is detected, a facsimile request for randomization will be send to the consortium leader. The 1:1 randomization is performed by authorized study personnel of the University Medical Center Hamburg-Eppendorf for each site using a blinded computer-generated randomization-list to either the intervention or the control group. The result of the randomization will be documented and send back to the site via facsimile. To achieve a rapid response and ensure smooth communication, a telephone is configured especially for the randomization procedure within the CFC-P.

### Ethics

All local ethics committees in the consortium approved the study protocol. The leading ethics committee is the Hamburg Medical Chamber. Local ethics committees are the „Ethikkommission an der Medizinischen Fakultät der Rheinischen Friedrich-Wilhelms-Universität Bonn “belonging to University Hospital Bonn, „Ethikkommission der Friedrich-Alexander-Universität Erlangen-Nürnberg “belonging to University Hospital Erlangen, „Ethik-Kommission der Medizinischen Fakultät der Universität Duisburg-Essen" belonging to University Hospital Essen, „Ethik-Kommission der Albert-Ludwigs-Universität Freiburg “belonging to Medical Center University of Freiburg, „Ethik-Kommission der Medizinischen Hochschule Hannover “belonging to Hannover Medical School, „Ethik-Kommission der Friedrich-Schiller-Universität Jena “belonging to University Hospital Jena, „Ethikkommission der Universität zu Lübeck” belonging to University Hospital of Schleswig-Holstein, Campus Lübeck, “Ethik-Kommission der Otto-von-Guericke-Universität an der Medizinischen Fakultät und am Universitätsklinikum Magdeburg A. ö. R. “belonging to Medical Faculty University Hospital Magdeburg, „Ethikkommission der Landesärztekammer Rheinland-Pfalz K.d.ö.R. “belonging to Mainz University Medical Center, „Ethik-Kommission der Ärztekammer Westfalen-Lippe und der Westfälischen Wilhelms-Universität Münster “belonging to University Children’s Hospital Münster, „Ethikkommission an der Medizinischen Fakultät der Universität Rostock “belonging to University Hospital Rostock, „Ethik-Kommission bei der Landesärztekammer Baden-Württemberg “belonging to Olgahospital Stuttgart and „Ethik-Kommission bei der Medizinischen Fakultät der Universität Würzburg, Institut für Pharmakologie “belonging to University Hospital Würzburg.

The study is conducted in accordance with the Declaration of Helsinki, Good Clinical Practice guidelines, including data and patient’s privacy protection. All participants provide written informed consent. The CFC-P was registered on 19th January 2018 prospectively and received the ID DRKS00012504. Recruitment has started in January 2018.

### Statistical methods

All analyses will be performed in accordance with the intention-to-treat principle. The first primary endpoint “Rate of CAYAs with need for intervention after 12 months” will be compared using a likelihood-ratio Chi^2^ test. The co-primary endpoint “Rate of CAYAs with needs not yet covered in the assessment” will only be tested if the null-hypothesis for the first primary endpoint is rejected (hierarchical testing). The closed testing procedure of Lehmacher et al. will be applied [88].

Effects will be reported as absolute and relative risk changes with 95% confidence intervals.

### Sample size calculation

Primary endpoint (rate of CAYAs with need for intervention after 12 months).

Within the group with high needs it is expected that basic care will reduce the need for interventions by 10%. The need-stratified interventions of the CFC-P should reduce the need for interventions by additional 15 to 75% after 12 months. Using the likelihood ratio Chi^2^ test and taking into account an alpha value of 5% and a beta error of 10%, 242 CAYAs must complete the 12-month evaluations. Considering a drop-out rate of approximately 30%, a total of 350 CAYAs with initial high needs will be 1:1 randomized to basic care or need-based interventions. It is expected that about 60% of CAYAs will have needs that require intervention, thus 530 CAYAs have to be recruited for the randomized phase. The programme will continue afterwards, and it is planned to include overall 1500 participants in this three-year time frame.

### Consent

Patients deemed eligible for entry into the study will be provided with a verbal and written explanation of the study. After adequate time has been given, and all queries have been addressed and the clinical team is confident that the patient understands the study, patients will be asked to consent to the study. The written declaration of consent of minor participants (under 18) needs to be signed a parent or guardian.

### Data collection and confidentiality

Confidentiality (with regard to the Federal Data Protection Act) of all patient-related data is ensured as all data will be pseudonymously (encrypted) stored and evaluated. A separate log relating original patient data with its respective, encrypted data will be created and appropriately secured by password and only authorized study personnel will be granted access to this file. Each investigator must ensure the patients’ confidentiality is maintained. Information and measurements of the study participants collected during the study will be recorded and stored separately from the personal information. Immediately after data collection, the data will be pseudonymously stored via the patient ID. All collected data will remain in secured locations and servers. The written and documented personal data, as well as the illness or health information, will be sealed and stored separately from each other.

### Access to data

The responsible investigators commit to archive all documents of the study for 15 years after the completion of the study.

## Discussion

Multimodal cancer treatment, including surgery, radiotherapy, chemotherapy, immunotherapy, allogeneic HSCT and/or endocrine or targeted therapy can result in relevant long-term sequelae. CAYAs face significant, partly severe and at times life-threatening late effects that can affect different organs e.g. endocrine system, heart, bones, cognitive and neurological system and can cause secondary malignancies. Furthermore, CAYAs have a high rate of unmet psychosocial needs that are currently neither regularly assessed nor cared for [89]. Although CAYAs have faced a severe life-threatening disease in the early years of their lives, one third of survivors have a risky health behaviour and an unhealthy lifestyle [32]. CAYAs have a moderate to poor nutritional behaviour and are insufficiently active compared to controls. Therefore, improving the lifestyle behaviour of CAYAs is important to reduce the risk for cardiovascular long-term toxicities in particular. Individualized exercise and nutrition interventions to promote physical activity and a healthy diet are needed after cancer treatment in order to enhance the lifestyle of CAYAs. So far only a few randomized intervention trials have examined the physical activity or nutritional behaviour of CAYAs. Supervised interventions containing a physical activity-educational and/or exercise intervention in a group setting improved physical activity, quality of life, cardiovascular, physical and metabolic outcomes of cardiovascular diseases [57, 58]. Interventions focusing on physical activity or healthy diet of young cancer survivors are practical, feasible and generally well accepted by the participants [54, 55, 90].

This randomized controlled multicentre trial will use a complex approach with the focus on three module-interventions: physical activity, nutrition and psycho-oncology. All interventions are supported by diverse tools, such as individual counselling, wearable activity monitoring, bio impedance analysis, training and cooking classes, regular newsletters about healthy lifestyle, also, optionally, an anamnesis of smell and taste and spiroergometry. The counselling about physical activity and/or nutrition will focus on overcoming CAYAs barriers for healthy behaviour. The CFC-P is the first randomized trial with young cancer survivors to apply motivational interviewing (ono-to-one sessions) within the psycho-oncology module.

The results of this study will show whether the targeted interventions can reduce the rate of CAYAs with unmet needs at 12 month, the feasibility of a comprehensive lifestyle survivorship programme and the efficacy of modular interventions e.g. the individual need, cardiovascular risk factors and quality of life or fatigue at 12 months in relation to the initial assessment.

In conclusion, comprehensive cancer care has to include more than medical tumour follow-up, particularly in CAYAs. Clinicians should be aware of this vulnerable group of patients for better detection, prevention, and management of treatment-induced late effects. Follow-up care should be undertaken by a team of specialists with different disciplines, including paediatrics and medical oncologists, psycho-oncologists, endocrinologists, cardiologists, social workers, specialists for nutrition, sport scientists and others. Besides the treatment of any side effects, regular assessment and detection of early signs of potential problems or disorders and related preventative interventions should be one of the main issues in follow-up care.

Thus, the CFC-P was designed to establish a follow-up care programme for CAYAs at 15 large sites in Germany, to be implemented at further sites upon demonstration of the efficacy of the programme. Results of the CFC-P are expected by the end of 2020. During the final phase of the programme the results will be evaluated and discussed with health care insurances to ensure continuation of the programme within the standard of care. The two major health care insurances in Germany (AOK Rheinland/Hamburg and TK) are partners of the programme and all interventions were developed based on future standard of care accounting.

## Data Availability

The data that will be generated and analysed during the current study is not publicly available due to the sensitivity of the collected data. The data or parts of the data will be available from the corresponding author on reasonable request.

## Abbreviations

ALL: Acute lymphoblastic leukaemia
AYA: Adolescents and Young Adults
BIA: Bio impedance analysis
BMI: Body Mass Index
BSA: Physical Activity, Exercise and Sport Questionnaire
CAYA: Children, adolescents and young adults
CBT: Cognitive Behavioural Therapy
CCSS: Childhood Cancer Survivor Study
CFC-P: CARE for CAYA-Program
DGE: The German Nutrition Society
EGFR: Epidermal growth factor receptor
EORTC QLQ C30: European Organisation for Research and Treatment of Cancer Quality of Life Questionnaire Core 30
HADS-D: Hospital Anxiety and Depression Scale
HEI-EPIC: Healthy Eating Index of the European Prospective Investigation into Cancer and Nutrition (EPIC)-Potsdam-Study
HSCT: Haematopoietic stem cell transplantation
INAYA: Improved Nutrition in AYAs
MET: Metabolic equivalent
MI: Motivational Interviewing
NCCN: National Comprehensive Cancer Network
PHQ-4: Patient Health Questionnaire-4
PTSD: Posttraumatic stress disorder
TTM: Transtheoretical Model
WHR: Waist-Hip Ratio

## Footnotes

1 Database Query of the Robert Koch Institute (www.krebsdaten.de), May 2019.

2 Extended definition based on The National Cancer Institute, who has defined the age range of adolescent and young adult cancer patients (AYAs) as being from 15 to 39 years (https://www.cancer.gov/types/aya).

## References

[1] Creutzig U (2003). Krebserkrankungen bei Kindern - Erfolg durch einheitliche Therapiekonzepte seit 25 Jahren. Deutsches Ärzteblatt.

[2] Robison LL, Hudson MM (2014). Survivors of childhood and adolescent cancer: life-long risks and responsibilities. Nat Rev Cancer.

[3] Krebs in Deutschland 2011/2012 (2015). Robert Koch-Institut (Hrsg) und die Gesellschaft der epidemiologischen Krebsregister in Deutschland e.V. (Hrsg): Berlin.

[4] Pritzkuleit R (2014). Auswertung der Krebshäufigkeit für die Leitlinie “Heranwachsende und junge Erwachsene (AYA, Adolescents and Young Adults)”.

[5] Oeffinger KC (2006). Chronic health conditions in adult survivors of childhood cancer. N Engl J Med.

[6] Rose SR (2016). Late endocrine effects of childhood cancer. Nat Rev Endocrinol.

[7] Stava CJ, Jimenez C, Vassilopoulou-Sellin R (2007). Endocrine sequelae of cancer and cancer treatments. J Cancer Surviv.

[8] Oeffinger K, Hudson M (2004). Long-term Complications Following Childhood and Adolescent Cancer. CA Cancer J Clin.

[9] Diller L (2009). Chronic disease in the childhood Cancer survivor study cohort: a review of published findings. J Clin Oncol.

[10] Mulrooney DA (2009). Cardiac outcomes in a cohort of adult survivors of childhood and adolescent cancer: retrospective analysis of the Childhood Cancer Survivor Study cohort. BMJ.

[11] van der Pal HJ (2012). High risk of symptomatic cardiac events in childhood cancer survivors. J Clin Oncol.

[12] Lipshultz SE (1991). Late cardiac effects of doxorubicin therapy for acute lymphoblastic leukemia in childhood. N Engl J Med.

[13] Lustig RH (2003). Octreotide therapy of pediatric hypothalamic obesity: a double-blind, placebo-controlled trial. J Clin Endocrinol Metab.

[14] Zhang FF (2015). Obesity is an important health problem in survivors of pediatric acute lymphoblastic leukemia. Pediatr Blood Cancer.

[15] Taskinen M (2000). Impaired glucose tolerance and dyslipidaemia as late effects after bone-marrow transplantation in childhood. Lancet.

[16] Meacham LR (2009). Diabetes mellitus in long-term survivors of childhood cancer. Increased risk associated with radiation therapy: a report for the childhood cancer survivor study. Arch Intern Med.

[17] Mostoufi-Moab S (2012). Longitudinal assessment of bone density and structure in childhood survivors of acute lymphoblastic leukemia without cranial radiation. J Clin Endocrinol Metab.

[18] Cohen LE (2012). Bone density in post-pubertal adolescent survivors of childhood brain tumors. Pediatr Blood Cancer.

[19] Chemaitilly W (2018). Endocrine late effects in childhood Cancer survivors. J Clin Oncol.

[20] HAVIGHURST ROBERT J. (1973). History of Developmental Psychology: Socialization and Personality Development through the Life Span. Life-Span Developmental Psychology.

[21] Zebrack BJ (2011). Psychological, social, and behavioral issues for young adults with cancer. Cancer.

[22] Eiser C, Aura K (2007). Psychological support for adolescents and young adults. Cancer in Adolescents and Young Adults. Cancer in Adolescents and Young Adults.

[23] Zebrack BJ, Penn A (2007). Psychosocial support. Cancer in adolescents and young adults.

[24] Albritton K, Bleyer WA (2003). The management of cancer in the older adolescent. Eur J Cancer.

[25] Prasad PK (2015). Psychosocial and neurocognitive outcomes in adult survivors of adolescent and early young adult Cancer: a report from the childhood Cancer survivor study. J Clin Oncol.

[26] Parsons HM (2012). Impact of cancer on work and education among adolescent and young adult cancer survivors. J Clin Oncol.

[27] Bhatt NS (2019). Post-transplantation employment status of adult survivors of childhood allogeneic hematopoietic cell transplant: a report from the Center for International Blood and Marrow Transplant Research (CIBMTR). Cancer.

[28] Barrera M (2005). Educational and social late effects of childhood cancer and related clinical, personal, and familial characteristics. Cancer.

[29] Kirchhoff AC (2012). Marriage and divorce among young adult cancer survivors. J Cancer Surviv.

[30] Nipp RD (2017). Financial burden in survivors of childhood Cancer: a report from the childhood Cancer survivor study. J Clin Oncol.

[31] de Fine Licht S, Rugbjerg K, Gudmundsdottir T (2017). Long-term inpatient disease burden in the Adult Life after Childhood Cancer in Scandinavia (ALiCCS) study: A cohort study of 21,297 childhood cancer survivors. PLoS Med.

[32] Klosky JL (2012). Risky health behavior among adolescents in the childhood cancer survivor study cohort. J Pediatr Psychol.

[33] Tao ML (1998). Smoking in adult survivors of childhood acute lymphoblastic leukemia. J Natl Cancer Inst.

[34] Haupt R (1992). Smoking habits in survivors of childhood and adolescent cancer. Med Pediatr Oncol.

[35] Hollen PJ (2007). Substance use risk behaviors and decision-making skills among cancer-surviving adolescents. J Pediatr Oncol Nurs.

[36] Marjerrison S (2016). Smoking, binge drinking, and drug use among childhood Cancer survivors: a meta-analysis. Pediatr Blood Cancer.

[37] Mayer DK (2007). Health behaviors in Cancer survivors. Oncol Nurs Forum.

[38] Quidde J (2016). Improved nutrition in adolescents and young adults after childhood cancer - INAYA study. BMC Cancer.

[39] Zhang FF (2015). Comparison of childhood cancer survivors' nutritional intake with US dietary guidelines. Pediatr Blood Cancer.

[40] Lown EA (2016). Patterns and predictors of clustered risky health behaviors among adult survivors of childhood cancer: a report from the childhood Cancer survivor study. Cancer.

[41] Murnane A (2015). Adolescents and young adult cancer survivors: exercise habits, quality of life and physical activity preferences. Support Care Cancer.

[42] Ranft A (2017). Quality of survivorship in a rare disease: Clinicofunctional outcome and physical activity in an observational cohort study of 618 Long-term survivors of Ewing sarcoma. J Clin Oncol.

[43] Gotte M (2015). Motor performance in children and adolescents with cancer at the end of acute treatment phase. Eur J Pediatr.

[44] Slater ME (2015). Physical activity, fitness, and Cardiometabolic risk factors in adult survivors of childhood Cancer with a history of hematopoietic cell transplantation. Biol Blood Marrow Transplant.

[45] Robinson PD (2018). Management of fatigue in children and adolescents with cancer and in paediatric recipients of haemopoietic stem-cell transplants: a clinical practice guideline. Lancet Child Adolesc Health.

[46] Jones LW (2014). Exercise and risk of major cardiovascular events in adult survivors of childhood hodgkin lymphoma: a report from the childhood cancer survivor study. J Clin Oncol.

[47] Scott JM (2018). Association of Exercise with Mortality in adult survivors of childhood Cancer. JAMA Oncol.

[48] Howell CR (2018). Clinical impact of sedentary behaviors in adult survivors of acute lymphoblastic leukemia: a report from the St. Jude Lifetime Cohort study. Cancer.

[49] Mays D (2011). Efficacy of the survivor health and resilience education (SHARE) program to improve bone health behaviors among adolescent survivors of childhood cancer. Ann Behav Med.

[50] Kaste SC (2014). Calcium and cholecalciferol supplementation provides no added benefit to nutritional counseling to improve bone mineral density in survivors of childhood acute lymphoblastic leukemia (ALL). Pediatr Blood Cancer.

[51] Jarvela LS (2016). Home-based exercise training improves left ventricle diastolic function in survivors of childhood ALL: a tissue Doppler and velocity vector imaging study. Pediatr Blood Cancer.

[52] Le A (2017). A home-based physical activity intervention using activity trackers in survivors of childhood cancer: a pilot study. Pediatr Blood Cancer.

[53] Blaauwbroek R (2009). The effect of exercise counselling with feedback from a pedometer on fatigue in adult survivors of childhood cancer: a pilot study. Support Care Cancer.

[54] Berg CJ (2014). Pilot results of an online intervention targeting health promoting behaviors among young adult cancer survivors. Psychooncology.

[55] Rabin C (2011). Internet-based physical activity intervention targeting young adult Cancer survivors. J Adolesc Young Adult Oncol.

[56] Valle CG (2013). A randomized trial of a Facebook-based physical activity intervention for young adult cancer survivors. J Cancer Surviv.

[57] Long TM (2018). Exercise training improves vascular function and secondary health measures in survivors of pediatric oncology related cerebral insult. PLoS One.

[58] Keats MR, Culos-Reed SN (2008). A community-based physical activity program for adolescents with cancer (project TREK) - program feasibility and preliminary findings. J Pediatr Hematol Oncol.

[59] Rouleau CR, Garland SN, Carlson LE (2015). The impact of mindfulness-based interventions on symptom burden, positive psychological outcomes, and biomarkers in cancer patients. Cancer Manag Res.

[60] Pinto BM, Floyd A (2008). Theories underlying health promotion interventions among cancer survivors. Semin Oncol Nurs.

[61] Spencer JC, Wheeler SB (2016). A systematic review of motivational interviewing interventions in cancer patients and survivors. Patient Educ Couns.

[62] Rollnick S (2010). Motivational interviewing. BMJ.

[63] Garrett K (2013). Bridging the transition from cancer patient to survivor: pilot study results of the Cancer survivor telephone education and personal support (C-STEPS) program. Patient Educ Couns.

[64] Ream E (2015). Management of cancer-related fatigue during chemotherapy through telephone motivational interviewing: modeling and randomized exploratory trial. Patient Educ Couns.

[65] Allicock M (2014). Implementing a one-on-one peer support program for cancer survivors using a motivational interviewing approach: results and lessons learned. J Cancer Educ.

[66] Djuric Z (2011). A diet and exercise intervention during chemotherapy for breast Cancer. Open Obes J.

[67] Spector D (2014). A pilot study of a home-based motivational exercise program for African American breast cancer survivors: clinical and quality-of-life outcomes. Integr Cancer Ther.

[68] Saloustros E (2017). The care of adolescents and young adults with cancer: results of the ESMO/SIOPE survey. ESMO Open.

[69] Cannioto R (2016). Chronic recreational physical inactivity and epithelial ovarian Cancer risk: evidence from the ovarian Cancer association consortium. Cancer Epidemiol Biomark Prev.

[70] Ruesten AV (2009). Die Bewertung der Lebensmittelaufnahme mittels eines ,Healthy Eating Index‘(HEI-EPIC). Ernährungs Umschau.

[71] Guenther PM (2013). Update of the healthy eating index: HEI-2010. J Acad Nutr Diet.

[72] Promotion, C.f.N.P.a (1995). USDoA: The Healthy Eating Index.

[73] Anja Mehnert DM (2006). Claudia Lehmann und Uwe Koch, Die deutsche VersiondesNCCN Distress-Thermometers. Z Psychiatr Psychol Psychother.

[74] Lowe B (2010). A 4-item measure of depression and anxiety: validation and standardization of the patient health Questionnaire-4 (PHQ-4) in the general population. J Affect Disord.

[75] Krämer L, Fuchs R (2009). Skalen zu den sportbezogenen situativen Barrieren und dem sportbezogenen Barrieremanagement.

[76] DiClemente CC, Prochaska JO (1982). Self-change and therapy change of smoking behavior: a comparison of processes of change in cessation and maintenance. Addict Behav.

[77] Buchholz D (2015). Entstehung und Definitionen. Manual für den German-Nutrition Care Process (G-NCP).

[78] Aaronson NK (1993). The European Organization for Research and Treatment of Cancer QLQ-C30: a quality-of-life instrument for use in international clinical trials in oncology. J Natl Cancer Inst.

[79] Löllgen, Das Anstrengungsempfinden (RPE, Borg-Skala) (2004). Deutsche zeitschrift für sportmedizin.

[80] Fuchs R (2015). Messung der Bewegungs- und Sportaktivität mit dem BSA-Fragebogen. Zeitschrift für Gesundheitspsychologie.

[81] Schmidt J, Lamprecht F, Wittmann WW (1989). Satisfaction with inpatient Care development of a questionnaire and 1st validity assessments. Psychother Psychosom Med Psychol.

[82] Herdmann M (2011). Development and preliminary testing of the new five-level version of EQ-5D (EQ-5D-5L). Qual Life Res.

[83] Egger N (2015). Short-term cost-effectiveness of psychodynamic therapy and cognitive-behavioral therapy in social anxiety disorder: results from the SOPHO-NET trial. J Affect Disord.

[84] Chisholm D (2000). Client Socio-Demographic and Service Receipt Inventory--European Version: development of an instrument for international research. EPSILON Study 5. European Psychiatric Services: Inputs Linked to Outcome Domains and Needs. Br J Psychiatry Suppl.

[85] Eigenentwicklung universitäres cancer center, U., Inanspruchnahme medizinischer Leistungen, Auszug Stand 03.11.2017.

[86] Eigenentwicklung Institut für medizinische Psychologie, U., Inanspruchnahme psychoonkologischer Leistungen, Auszug. Stand 03.11.2017.

[87] Girgis A (2012). The next generation of the supportive care needs survey: a brief screening tool for administration in the clinical oncology setting. Psychooncology.

[88] Lehmacher W, Wassmer G, Reitmeir P (1991). Procedures for two-sample comparisons with multiple endpoints controlling the experimentwise error rate. Biometrics.

[89] Cox CL (2016). The unmet emotional, care/support, and informational needs of adult survivors of pediatric malignancies. J Cancer Surviv.

[90] Howell Carrie R., Krull Kevin R., Partin Robyn E., Kadan-Lottick Nina S., Robison Leslie L., Hudson Melissa M., Ness Kirsten K. (2018). Randomized web-based physical activity intervention in adolescent survivors of childhood cancer. Pediatric Blood & Cancer.

